# Fine-grain recordings of the electrically evoked compound action potential amplitude growth function in cochlear implant recipients

**DOI:** 10.1186/s12938-018-0588-z

**Published:** 2018-10-19

**Authors:** Lutz Gärtner, Thomas Lenarz, Andreas Büchner

**Affiliations:** Department of Otolaryngology, Hannover Medical School, Carl-Neuberg-Str. 1, 30625 Hannover, Germany

**Keywords:** ECAP, Electrically evoked compound action potential, Cochlear implant, Amplitude growth function, Fine-grain

## Abstract

**Background:**

In cochlear implants (CI) measuring the electrically evoked compound action potential (ECAP) has become an important tool for verifying the electrode-nerve interface as well as establishing a basis for a map to program the speech processor. In a standard clinical setup recordings are averaged over 25–100 repetitions to allow for the detection of ECAPs within the noise floor. To obtain an amplitude growth function, these measurements are normally performed for 5–10 different stimulation levels. We evaluate a recording paradigm where the stimulation intensity is increased in quasi-continuous steps and instead of averaging repeated recordings with identical stimulation parameters, running averages over small intervals of stimulation levels are computed. The first visible nerve response was manually identified by two experts.

**Results:**

Both recording paradigms were evaluated in 39 cochlear implants, showing an on average lower threshold of the first nerve response for the quasi-continuous measurement paradigm (Wilcoxon signed-rank test, p = 6.2e−08) compared to the clinical standard paradigm. The mean maximal loudness over all implants and stimulation electrodes was 13% lower at the 80 pulses/s quasi-continuous paradigm compared to the 44 pulses/s clinical standard paradigm.

**Conclusions:**

Beside a more robust determination of the ECAP threshold, the proposed quasi-continuous stimulation paradigm results in a more robust behavioral feedback of the CI user upon the maximal acceptable loudness percept. Furthermore this paradigm can also reveal the fine-structure in the amplitude growth function.

## Background

**Fig. 1:**
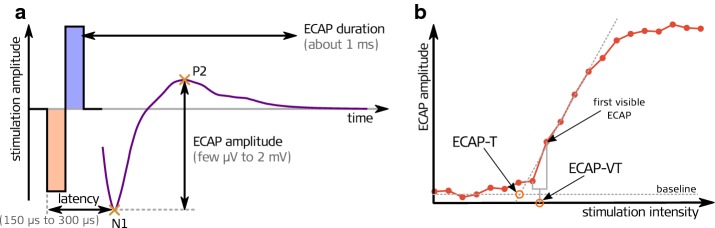
The left panel (**a**) depicts a representative ECAP waveform after biphasic stimulation. Note the negative peak N1 and the second positive peak P2, whose amplitude difference define the ECAP amplitude. The right panel (**b**) shows an ECAP amplitude growth function that normally exhibits a sigmoidal shape. The threshold of the ECAP amplitude growth function (ECAP-T) is normally defined as the crossing of the linear extrapolation in the inflection point of the sigmoid with the baseline. The estimation of the ECAP threshold can also be performed visually (ECAP-VT), being the stimulation level between the stimulation intensities before and when a first visible ECAP can be observed

**Fig. 2:**
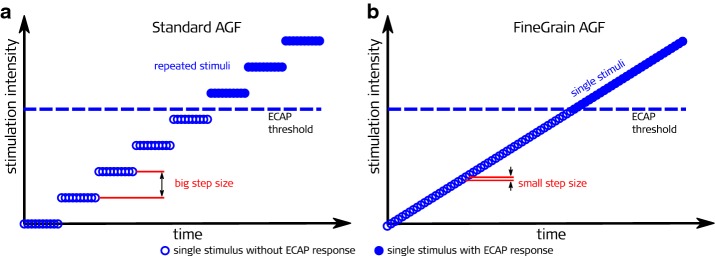
Comparison of the standard clinical and the quasi-continuous fine-grain paradigm. The number of single stimuli and time needed to record an AGF was the same for both paradigms. **a** (left): Averaging over several repetitions and big step size of stimulation amplitude leads to a coarse AGF; **b** (right): Single stimuli and considerably smaller step size leads to a fine-grain AGF

Cochlear implants (CI) are successful auditory prostheses used to restore hearing in deaf people. They consist of an electrode array with several electrode contacts, which is normally inserted into the scala tympani. Electrical stimulation on distinguished electrode contacts will evoke an action potential in the surrounding neural populations and will finally lead to an acoustic sensation. Intra- and post-operatively, the integrity of the electrode-nerve interface can be proven by recording the electrically evoked compound action potential (ECAP) which shows a characteristic response with a negative peak and one or two positive peaks [1], see Fig. 1a. The ECAP amplitude, defined as the difference between the electric potential of the positive and the negative peak, will be detectable above a certain threshold level and will rise with increasing stimulation intensity, thereby describing a so-called amplitude growth function (AGF) (see Fig. 1b). All manufacturers of cochlear implants provide the means to record ECAP AGF via the inserted electrode in commercially available, clinical programming software. To reduce the noise floor, the recordings are usually averaged over 15–100 repetitions. To keep the measurement time within a practical range, e.g. below 10–15 min, the AGF is usually recorded with 5–10 different stimulation levels in standard clinical setups (Fig. 2a).

The estimation of the ECAP threshold can be done in multiple ways. Using the information of the ECAP amplitude growth function, the threshold of the AGF can be defined as the crossing of the linear extrapolation in the inflection point of the sigmoid with the baseline (ECAP-T, see Fig. 1b). Using a visual detection of responses in single ECAP recording, a visual threshold (ECAP-VT) can be defined as the stimulation intensity between the highest stimulation intensity where no response is visible and the lowest stimulation intensity where a response can be detected. With this approach the maximal error in estimating the threshold is half the step size of the different subsequently applied stimulation levels. The error can be minimalized by reducing the step size but thereby increasing the measurement time.

We evaluate a recording paradigm where the stimulation intensity is increased in quasi-continuous steps and instead of averaging repeated recordings with identical stimulation parameters, moving averages over small intervals of approximately identical stimulation levels are computed. The total number of single ECAP recordings is identical, resulting in identical measurement durations. The aim of this study was on the one hand to investigate if the new approach is feasible and safe for recipients in postoperative measurements. The other goal of the study was to analyze whether a moving average over adjacent different stimulation levels can be used to reduce the noise floor while leading to thresholds which are comparable to those found with the standard clinical method.

## Results

The number of AGFs recorded for each paradigm was 468 (39 implants with 12 electrode contacts). 459 electrode contacts were activated during the fitting procedure. ECAPs were recorded also from electrodes that were not activated. In 356 cases the human experts could determine visually an ECAP threshold. In each recording every recipient could indicate in time when the sound percept was getting too loud. No recipient complained about a sudden or too fast rise in loudness.

**Fig. 3:**
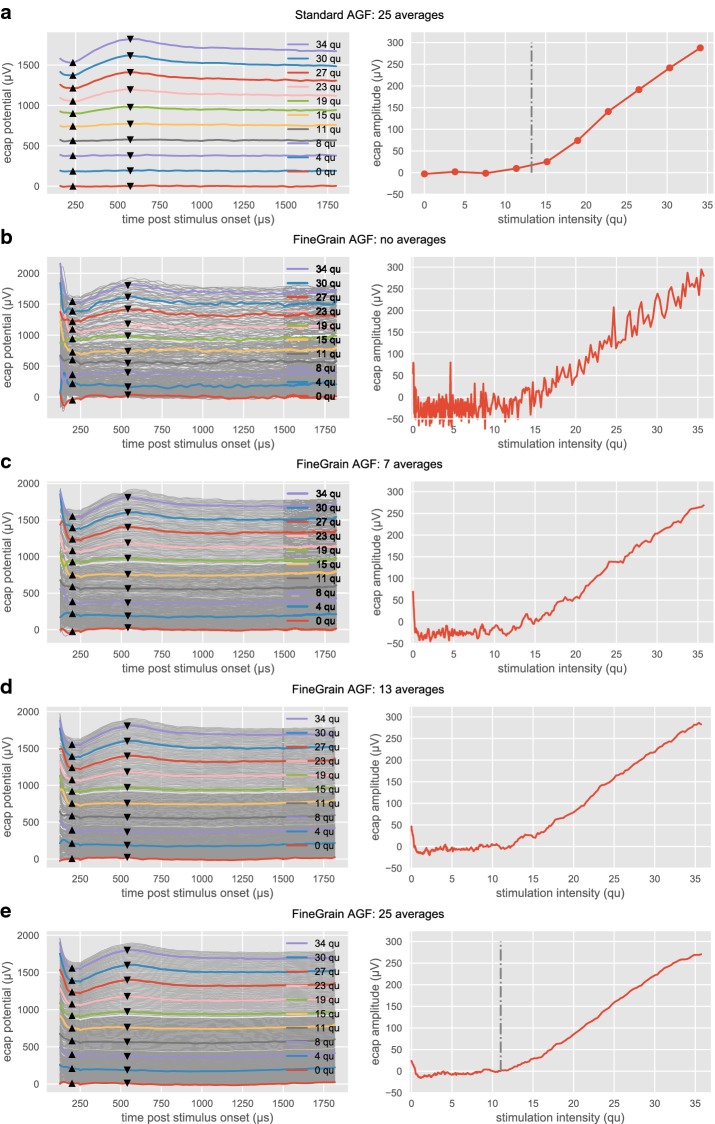
Example of AGF recordings. Fixed latencies were used for N1 (233 µs) and P2 (575 µs). The left columns show the single ECAP waveforms as a waterfall plot. ECAP waveforms with matching stimulation intensity are marked with identical colors. For better readability, the large amount of available ECAP waveforms in the FineGrain AGF paradigm is plotted in gray. Note that always the full dataset is processed to generate the quasi-continuous amplitude growth function shown on the right columns. Row **a** shows the paradigm Standard AGF (25 averages per stimulation level). Row **b** paradigm FineGrain AGF (without moving average) and **c**–**e** with a moving average over 7, 13 and 25 different stimulation levels, respectively. For row **a**, **e**, the mean visual ECAP threshold is plotted as a dash-dotted line

An example of the recordings with the two paradigms and different moving averages is shown in Fig. 3. To plot the AGF, fixed latencies according to [2] were used for the N1 peak (233 µs) and N2 peak (575 µs).

Figure 3a represents recordings for the Standard AGF paradigm. The left pane shows ECAP recordings for ten different stimulation levels where each level was measured 25 times and the response was averaged over identical stimulation parameters. The characteristic N1 peak and P2 peak are visible for stimulation levels above 13 qu, being the visual threshold (ECAP-VT). The right pane shows the AGF where each measured ECAP amplitude (dots) versus stimulation level was connected with a straight line. The ECAP-VT is marked with a vertical dash-dotted line.

Figure 3b–e represent recordings for the FineGrain AGF paradigm. While Fig. 3b shows original data, in Fig. 3c–e a moving average was superimposed, where the number of recordings used for the moving average varied from 7 (n_−3_, n_−2_, n_−1_, n, n_1_, n_2_, n_3_) to 13 (n_−6_, …, n_6_) and 25 (n_−12_, …, n_12_) levels, respectively. The index n characterizes the consecutive number of the recording. In the left panes ECAP recordings are shown, where traces for stimulation intensities which match the recordings of Fig. 3a are plotted using the same color. To enhance the readability, the remaining fine-grain measurements are plotted as thin gray traces. Note that always the full dataset is used, resulting in quasi-continuous amplitude growth functions shown in the right panes.

**Fig. 4:**
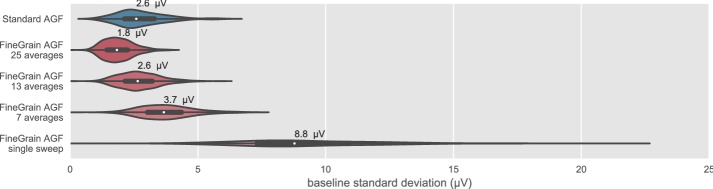
Noise level estimated for all 468 Standard AGF (blue) and 468 FineGrain AGF (red) via the standard deviation of the baseline at the end of an ECAP recording (1.3–1.6 ms after stimulus onset) after averaging. The median noise level is given above each group. Note that Standard AGF was measured with clinical software (MAESTRO 4.1.2) and FineGrain AGF with a research tool provided by MED-EL whose different averaging algorithm and respective low-pass characteristics are the main cause for the lower noise floor of FineGrain AGF at the same number of averages

Averaging over 25 repeated identical measurements results for all 468 stimulation electrodes into a median standard deviation of the baseline of 2.6 µV. (see Fig. 4). Single ECAP recordings without averaging had a median standard deviation of the baseline of 8.8 µV. Performing a moving average over 7, 13 and 25 ECAP recordings reduced the estimated noise to a median standard deviation of the baseline of 3.7, 2.6 and 1.8 µV, respectively.

**Fig. 5:**
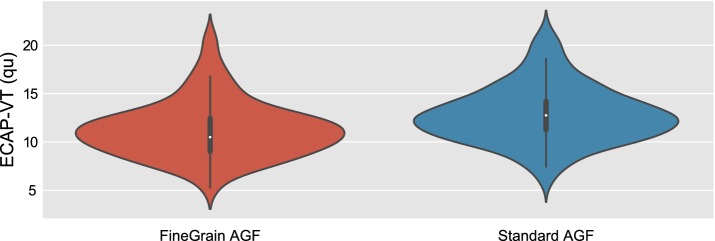
The visually estimated ECAP threshold (ECAP-VT) was significantly lower (Wilcoxon signed-rank test, p = 6.2e−08) for the FineGrain AGF paradigm compared to the Standard AGF

The distribution of the visually estimated ECAP thresholds (ECAP-VT) for each paradigm is shown in Fig. 5. The human experts evaluated in both cases ECAP waveforms that were averaged over 25 recordings. The ECAP-VT was significantly lower (Wilcoxon signed-rank test, p = 6.2e−08) for the FineGrain AGF paradigm compared to the Standard AGF.

**Fig. 6:**
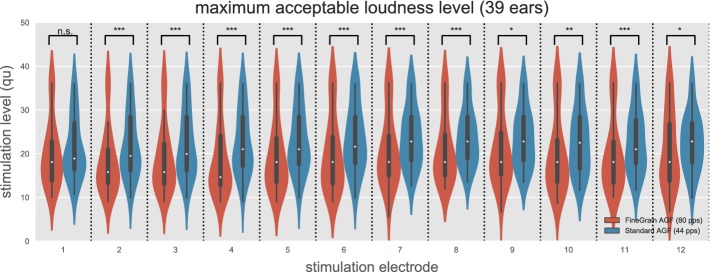
The maximum acceptable loudness level (MAL) for the Standard AGF paradigm (red) and the FineGrain AGF paradigm (blue). Except for the most apical electrode 1, all other electrodes showed a significant higher MAL within the Standard AGF paradigm. Note that the Standard AGF was measured at 44 pps compared to an 80 pps stimulus during the FineGrain AGF measurement paradigm. Different levels of significance are marked as: n.s. p ≥ 0.05, *p < 0.05, **p < 0.01, ***p < 0.001

The distribution of the maximum acceptable loudness level (MAL) for each electrode is shown in Fig. 6. Except for the most apical electrode 1, all other electrodes showed a significantly higher presented stimulus level within the 44 pps Standard AGF paradigm compared to the 80 pps stimulus during the FineGrain AGF measurement paradigm. Significance was tested via a two-sided *t*-test for the null hypothesis that two related samples have identical average expected values (for electrode 1–12 the p-values were 1.56e−1; 4.85e−6; 1.07e−5; 4.02e−6; 1.51e−5; 1.34−4; 7.03e−4; 1.75e−4; 1.05e−2; 1.91e−3; 1.21e−4; 3.32e−2). The mean MAL over all implants and stimulation electrodes was 13% lower at the 80 pps FineGrain AGF paradigm compared to the 44 pps Standard AGF measurements. No effect of the electrode array location on the magnitude of the MAL difference between the FineGrain AGF and Standard AGF was found (t-test, p > 0.7).

## Discussion

Several improvements to the standard amplitude growth sequence have been proposed. A variation of the Békésy tracking was proposed by [3], a binary search algorithm was presented by [4]. All these paradigms optimize the measured stimulation amplitudes, but keep the concept of averaging over identical stimulation amplitudes and thereby limit the total number of different stimulation levels to minimize the total measurement time. In this manuscript, we performed solely manual ECAP threshold determination by human experts to avoid any bias of the applied algorithm. Please see e.g. [2] for a recent discussion on the non-trivial task of ECAP threshold determination and a robust nerve response metric. Further research how the fine-grain recording paradigm affects different concepts of automatic threshold determination is needed.

The advantage of the information gain due to the quasi-continuous sampling can be seen in the context of the visual threshold (ECAP-VT) determination. For the AGF recorded with the standard paradigm (Fig. 3a, right pane), the baseline background activity can be estimated at approx. 15 µV from the measurements at 0, 4 and 8 qu. The response at 11 qu shows an elevated ECAP amplitude of 23 µV, but the morphology (left pane) exhibits no clear N1 or P2 peaks and both human experts labeled the first visible ECAP response at 15 qu with an ECAP amplitude of 36 µV. The ECAP-VT was therefore estimated at (15 qu + 11 qu)/2 = 13 qu. The FineGrain AGF in the right pane of Fig. 3e allowed the human experts to decide that the elevated response of 23 µV at 11 qu was already part of the neural response and did not belong to the noise floor. From a signal processing perspective, the Standard AGF can be understood as an undersampling of the space of all amplitude growth functions, suffering from a quantization error concerning the first above threshold stimulation amplitude.

The noise reduction shown in Fig. 4 follows the square root of N rule (see Table 1), hence can be mainly explained by the removal of uncorrelated noise.

**Table 1:** Effect of averaging on estimated noise

Number of averages	Estimated noise (µV)	Factor re single sweep	sqrt(N)
N = 7	3.7	2.5	2.6
N = 13	2.6	3.5	3.6
N = 25	1.9	4.8	5.0

The first column states the number of different stimulation amplitudes used for averaging. The second column shows the estimated noise. The third column shows the decrease in noise compared to the recording without any averaging. The forth column shows the expected reduction of uncorrelated noise due to the number of averages

Since the error in estimating the visual threshold (ECAP-VT) is given by half the step size, the lower visually estimated ECAP threshold found for the FineGrain AGF paradigm was inherent to the difference between the paradigms. With the relatively big step size of the Standard AGF paradigm the visual threshold will be over-estimated. Another factor to be considered is the improved estimation of the baseline activity by the human expert due to the denser sampling. This allows for an earlier visual detection when the response is above the standard deviation of the baseline (see also [2] for a more in-depth discussion).

With the FineGrain AGF paradigm the maximum acceptable loudness level (MAL) was lower compared to Standard AGF paradigm. One explanation could be the difference in stimulation rate. Stimulation rate has a known influence on loudness percept. Several studies have shown that electrical threshold is decreasing with increasing stimulation rate [5–8], but less studies have reported the effect of stimulation rate on MAL. To our knowledge no study in human CI users was conducted about the influence on MAL in the stimulation rate range of 40–80 pps with pulse durations of about 30 µs. Pfingst et al. [9] reported a small reduction of the behavioral threshold in normal hearing guinea pigs when increasing pulse rate from 39 to 78 pps while effects on the loudness percept in the deaf animal model were observed only for pulse rates above 1000 pps. In [5] it was shown, that the threshold is not affected by pulse rate for rates below 100 pps. Following the signal detection theory, an unchanged behavioural threshold can be explained by the same amount of available information, i.e. number of recruited nerve fibers. The ECAP amplitude directly correlates with the number of recruited nerve fibres, which supports the assumption, that the change in the ECAP threshold is not due to differences in the stimulation rate. In [10] it was shown that the ECAP threshold is reduced by about 2% comparing stimulation rates of 20 and 80 pps, with a variance of 2%. With an inter-pulse interval in the FineGrain AGF paradigm of 12.5 ms and 25 ms for the Standard AGF paradigm, the applied pulse rates were in both paradigms below the refractory time of the auditory nerve and no masking effect is to be expected. This suggests that the main difference in the loudness percept is not due to a different number of recruited nerve fibers.

**Fig. 7:**
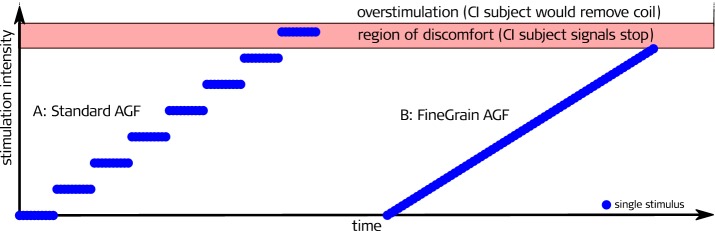
Assuming that the maximal acceptable loudness level is not a distinct level but a range, an AGF stair-case paradigm will jump into the region of discomfort, resulting in a higher loudest presented stimulation level compared to a quasi-continuous paradigm, which will always stop stimulation at the beginning of the region of discomfort

Another explanation for the different MAL could be the step size towards the region of discomfort (see Fig. 7). In the FineGrain AGF paradigm, the quasi-continuous increase results in an immediate stop when the region of the MAL is reached. Due to the larger step size in the Standard AGF paradigm, the stimulation where the subjects asked to stop is likely above the lower limit of the MAL. The Fine-Grain AGF paradigm continuously approaches the limit and thereby never surpasses it, which could explain the lower maximal stimulation level, but also indicates that this paradigm is safer in terms of the risk of overstimulation.

As the order of electrodes was not randomized, and the first stimulated electrode showed a non-significant difference of 4%, a build-up-effect could be another possible explanation for the different maximal stimulation intensity.

As the ECAP threshold is clinically used to help with the determination of an initial fitting map [11, 12], further research is needed to evaluate if the observed improved accuracy of the first visible ECAP threshold determination has any effect on the quality of the initial ECAP based map. It is to note that the general correlation between the ECAP threshold and the fitting parameter is intermediate and that there exist better objective measurements like the stapedius reflex [13]. The effect on other ECAP-based clinical application as e.g. described in [14] is also to be investigated.

## Conclusion

The intention of the study was to prove whether the new approach (FineGrain AGF) is feasible and safe and leads to comparable threshold values in comparison to the clinical software (Standard AGF). This was the case for each of the 39 implants in all 35 subjects.

The error of visual ECAP threshold estimation is half the step size and a smaller step size should therefore lead to a smaller estimation error. As the visual ECAP threshold estimation is a detection task, the first above threshold response will be reported leading to an always positive estimation error, i.e. an overestimation of the true threshold. The lower visual ECAP threshold for the FineGrain AGF paradigm compared to the Standard AGF (see Fig. 5) indicates that the new approach (FineGrain AGF) allows for a more accurate ECAP threshold estimation. Besides the clinical relevant ECAP threshold detection, in the context of research, the finer resolution of the stimulation amplitudes offers also possibilities to investigate the stimulated neural population of the auditory nerve. Usually the AGF is a monotonically ascending function. The authors found in rare cases an AGF with segments of different slopes and even with a negative slope [15]. By means of a fine grain AGF more of those cases might be discovered and analyzed.

## Methods

The study group comprises 35 adult CI users (18 male, 17 female) which have been chosen without any special consideration. In these recipients, measurements have been done with 39 implants, 5 MED-EL SONATAti100, and 34 MED-EL CONCERTO. Demographic data is summarized in Table 2.

**Table 2:** Demographic data of the study group

Pat-ID	Gender	Implant-ID	Age at implantation (years)	Implanted side	Implant and electrode type	Duration of CI in use at date of measurement (months)	Etiology: cause of deafness
R-01	F	CI-01	68.6	R	C Std	23.98	Unknown
R-02	M	CI-02	48.0	L	C Std	0.03	Unknown
R-03	M	CI-03	84.1	L	C 28	0.07	Sudden hearing loss
R-04	F	CI-04	48.0	R	C Std	0.07	Unknown
R-05	F	CI-05	71.8	R	C Std	36.37	Sudden hearing loss
R-06	M	CI-06	69.7	R	C 28	5.82	Sudden hearing loss
R-07	F	CI-07	60.2	R	C 28	0.03	Sudden hearing loss
R-08	M	CI-08	40.9	R	C Std	6.11	Brain damage in early Childhood
R-09	F	CI-09	58.2	R	C Std	6.57	Unknown
R-10	F	CI-10	64.1	R	C 28	25.76	Sudden hearing loss
R-11	F	CI-11	35.1	L	S Std	51.84	Unknown
R-12	F	CI-12	50.7	R	C 24	12.06	Sudden hearing loss
R-13	M	CI-13	58.6	L	C 28	5.98	Unknown
R-14	F	CI-14	78.9	R	C Std	2.99	Unknown
R-15	F	CI-15	44.1	L	C 28	12.19	Genetically
R-16	F	CI-16	63.6	R	C Std	24.02	Unknown
R-17	F	CI-17	76.0	R	C Std	24.48	Unknown
R-18	M	CI-18	54.0	R	C Std	0.03	Mumps
R-19	M	CI-19	58.9	L	C Std	34.79	Sudden hearing loss
R-19	M	CI-20	61.8	R	C 28	0.10	Unknown
R-20	M	CI-21	41.7	L	C Std	36.24	Unknown
R-21	M	CI-22	53.4	R	C Std	0.03	Genetically
R-22	M	CI-23	62.8	L	C 28	3.02	Sudden hearing loss
R-23	F	CI-24	63.1	R	C Std	0.07	Otitis media
R-24	M	CI-25	59.2	L	S Std	54.34	Unknown
R-25	M	CI-26	76.2	L	S Std	51.84	Unknown
R-26	F	CI-27	57.7	R	C 28	0.03	Sudden hearing loss
R-27	M	CI-28	47.2	L	C 28	23.59	Cerebral infarction
R-27	M	CI-29	47.2	R	C 28	23.59	Unknown
R-28	F	CI-30	73.6	R	C 24	3.12	Unknown
R-29	M	CI-31	61.1	L	C 20	23.69	Genetically
R-30	F	CI-32	69.0	R	C 28	5.32	Genetically
R-31	M	CI-33	33.9	R	C 28	11.96	Otitis media
R-32	M	CI-34	75.8	L	C Std	31.34	Genetically
R-32	M	CI-35	78.4	R	C Std	0.13	Unknown
R-33	M	CI-36	69.3	R	S 20	48.13	Occupational noise-induced hearing impairment
R-33	M	CI-37	71.8	L	S 20	18.92	Unknown
R-34	M	CI-38	73.7	R	C Std	27.01	Sudden hearing loss
R-35	F	CI-39	62.4	R	C 28	2.99	Unknown

Side of implantation: L indicates left side, R right side. Type of implant and electrode: C indicates “CONCERTO”, S “SONATAti100”, followed by the electrode type (Std for Standard electrode, numbers indicate the length of the FLEX electrode)

With each of the 39 implants two measurement paradigms (Fig. 2) have been conducted postoperatively subsequently within approximately 1 h. Those measurements were performed during the clinical follow-ups to minimize the time subjects have to spend for additional study measurements.

First the standard clinical paradigm (“Standard AGF”, see Fig. 2a) was measured. The recipient’s implant was connected via a transmitting coil to a DIB II interface box (MED-EL Company, Innsbruck, Austria) which was connected via a serial port to a personal computer operating the clinical software Maestro Version 4.1.2. Using the ART task, monopolar biphasic charge balanced pulses with a phase duration of 30 µs were delivered at a stimulation rate of 44 pps (pulses per second) to the electrode contacts. The interphase gap was 2.1 µs. This was the fastest stimulation possible with the DIB II interface box. The stimulation level was increased from zero to the individual maximum acceptable loudness level (MAL) of each recipient, but not higher than a maximum charge of 36 qu (charge unit, 1 qu equals approximately 1 nC). A minimum of 10 different stimulation levels was aimed to be reached within each AGF. This was accomplished by setting the step size to 0.9–1.2 qu. Each stimulation level was presented 25 times. The ECAP potential was recorded on an adjacent electrode contact and averaged for each stimulation level. This procedure has been done subsequently for each of the 12 electrode array contacts.

In the new so called fine-grain paradigm (”FineGrain AGF”, see also Fig. 2b), the setup was the same as described above, but instead of a DIB II serial interface box the new MAX USB interface box (MED-EL Company, Innsbruck, Austria) and a research software was used. In addition, a higher stimulation rate of 80 pps was applied, since this was the highest rate possible with the MAX interface box. The stimulation level was also increased from zero to MAL (again, not higher than 36 qu), but each stimulation level was presented only once and 150–300 different stimulation levels were measured, depending on the respective MAL of the subject. The same pulse parameters and recording electrodes as described for the Standard AGF paradigm were used.

In both paradigms, the stimulation artifact was removed using an alternating stimulation approach. Each measurement was performed twice, with a cathodic/anodic and an anodic/cathodic stimulation pulse, respectively. The subsequent averaging allows the symmetric part of the stimulus artifact to cancel out while the polarity invariant part of the ECAP signal remains and uncorrelated noise is reduced. The recording artifact of the system, also called signature, was removed by subtracting a template recorded at zero stimulation amplitude. The last post-processing step in both paradigms was a low-pass filtering of the averaged waveform to reduce noise in the high-frequency region. Note that due to the clinical setup, different software implementations were used. The cut-off frequency of the clinical software was 18 kHz (Standard AGF) while it was 5 kHz for the running average implementation of the research tool (FineGrain AGF).

In the Standard AGF paradigm measurements were averaged over 25 repetitions of the same stimulus parameters to improve the signal-to-noise ratio. In case of the FineGrain AGF paradigm, a moving average was applied by taking into account the response evoked by a certain stimulation level, as well as up to 12 preceding and the following 12 levels, in total maximal 25 stimulation levels.

The noise of a single ECAP recording was estimated by computing the standard deviation of a 0.3 ms long analysis window, starting 1.3 ms after stimulus onset. The analysis window had thereby a distance of 0.1 ms to the end of the recording window. This region was chosen to contain no neural response and no filter artifacts. Note that noise and neural background activity will contribute to the sub-threshold baseline of an amplitude growth sequence (see Fig. 1b and [16]).

ECAP recordings were analyzed independently by two audiologists to estimate a visual threshold for both paradigms. The visual threshold (ECAP-VT) was defined as the average of the highest stimulation level without ECAP response and the lowest stimulation level that evoked an ECAP response (Fig. 1b). For both paradigms, an averaging over 25 recordings was performed.

During the measurements, the recipient was asked to indicate when her/his maximum acceptable loudness level (MAL) was reached so that the study operator could abort the measurement on the respective electrode contact and continue with the next scheduled stimulation electrode.

## Abbreviations

ECAP: electrically evoked compound action potential
CI: cochlear implant
AGF: amplitude growth function
MAL: maximum acceptable loudness level
QU: charge unit, 1 qu equals approximately 1 nC
ECAP-VT: first visual ECAP threshold
C: CONCERTO
S: SONATAti100
Std: standard
